# Role of some structural features in EPS from microalgae stimulating collagen production by human dermal fibroblasts

**DOI:** 10.1080/21655979.2023.2254027

**Published:** 2023-09-12

**Authors:** Claire Toucheteau, Valentine Deffains, Clément Gaignard, Christophe Rihouey, Céline Laroche, Guillaume Pierre, Olivier Lépine, Ian Probert, Didier Le Cerf, Philippe Michaud, Ingrid Arnaudin-Fruitier, Nicolas Bridiau, Thierry Maugard

**Affiliations:** a La Rochelle Université, UMR CNRS 7266 LIENSs, Equipe Biotechnologie et Chimie des Bioressources pour la Santé, La Rochelle, France; b Université Clermont Auvergne, CNRS, Institut Pascal, Aubière, France; c Université de Rouen Normandie, PBS Laboratory, Mont Saint Aignan, France; d Algosource Technologies, 37 Bd de l’Université, Saint-Nazaire, France; e Roscoff marine station, CNRS/Sorbonne Université, Roscoff, France

**Keywords:** Microalgal exopolysaccharide, depolymerization, high pressure pre-treatment, solid acid-catalyzed hydrolysis, human dermal fibroblast, procollagen effect, structure–function relationship

## Abstract

Exopolysaccharides (EPS) from the microalgae *Porphyridium cruentum*, *Chrysotila dentata*, *Pavlova* sp., *Diacronema* sp., *Glossomastix* sp., *Phaeodactylum tricornutum,* and *Synechococcus* sp. were isolated and depolymerized. First, EPS were submitted to a high pressure pre-treatment step, followed by a solid acid-catalyzed hydrolysis step carried out in a batch or recycle fixed-bed reactor, using a strong acidic cation-exchange resin. Twenty-eight different EPS forms were thus obtained. After characterization of their main structural features (weight- and number-averaged molecular weight, polydispersity index, sulfate and uronic acid contents), we investigated the structure–function relationship of their pro-collagen activity. We found that native microalgae EPS were able to inhibit until 27% of human matrix metalloproteinase-1 (MMP-1) activity while the depolymerized forms were able to enhance collagen production by two different human fibroblast lines, used as cell models due to their major role in dermal collagen biosynthesis. The most active EPS forms, obtained by depolymerization in the recycle fixed-bed reactor of *D. ennorea* and *Glossomastix* sp. EPS, led to 390% increase in collagen production. Finally, principal component (PCA) and Pearson analyses indicated that MMP-1 inhibition was strongly correlated to the sulfate group content of EPS whereas collagen production by fibroblasts was mostly related to their proportion of low molecular weight polysaccharides (<10 kDa). Uronic acid content of EPS was also shown essential but only if the size of EPS was reduced in the first place. Altogether, these results gave new insights of the dermo-cosmetic potential of microalgae EPS as well as the key parameters of their activity.

## Research Highlights

- Six new and original EPS from microalgae were isolated.
- Microalgae EPS were depolymerized by high pressure/solid acid-catalyzed hydrolysis.
- Native EPS inhibited human matrix metalloproteinase-1.
- Depolymerized EPS highly stimulated collagen production by human dermal fibroblasts.
- Structure–function studies revealed that sulfate groups and MW of EPS were crucial.

## 1. Introduction

Cutaneous aging is a normal complex biological phenomenon influenced by intrinsic and extrinsic factors, which are induced by normal cellular decline and environmental exposure to factors such as UV light [1]. Human skin aging has many consequences such as the remodeling of dermal extracellular matrix (ECM), inducing structural and composition modifications of ECM components, such as fibril collagen, elastin, or hyaluronic acid, as reflected by a sagging skin. Among these essential skin polymers, collagen, the most abundant protein of dermal ECM (90% of dry weight), is essentially responsible for the skin’s tensile strength and mechanical properties [2,3]. Type I and III collagens, assembled in a triple helix, are the main collagen forms found in the dermis. Collagen is produced in the skin by fibroblasts, which are the major cell types in the dermis and have an important role in preventing the formation of signs of skin aging, due to their capacity to produce several dermal ECM biopolymers, including collagen but also hyaluronic acid. During aging process, the proliferation and metabolic activities of dermal fibroblasts decrease, leading to the reduction of collagen biosynthesis and the appearance of signs of skin aging such as dehydration, atrophy, and loss of elasticity [4,5]. In a young skin, for example from a 20-year-old subject, collagen synthesis may start to decrease without signs of skin aging, compared to a more aged skin, whose rate of collagen production is drastically reduced and signs of skin aging can be obvious [6]. Furthermore, it has been shown very recently that collagen gene expression in human dermal fibroblasts progressively decreases with age [7].

For many years, the search for innovative natural ingredients has led to the development of many cosmetic products based on traditional marine sources, especially components derived from microalgae. These unicellular microorganisms are known to produce various compositions of biomolecules, such as proteins or lipids, depending on their species. Recently, microalgae have become particularly attractive due to their capacity to release polysaccharides into their environment, the so-called exopolysaccharides (EPS). EPS are polysaccharides unbound to the cell wall, generally produced by cyanobacteria, diatoms as well as red and green microalgae [8]. Less studied than EPS produced by other microorganisms such as bacteria, and much less than polysaccharides from marine macroalgae [9–11], they are generally polymers with a varied monosaccharide composition, like cell wall-bound polysaccharides, in spite of some exceptions such as *Gyrodinium impudicum* EPS that is made up of a single neutral monosaccharide, galactose, 3% uronic acid, and 10% sulfate groups [12]. One of the most well-known, the EPS from the marine microalga *Porphyridium cruentum*, is a highly sulfated acidic heteropolymer, exhibiting a molecular weight within the range 2000–7000 kDa [13,14]. This original polysaccharide has many biological activities such as antioxidant properties [15] or the capacity to inhibit two key enzymes involved in the remodeling of the ECM, elastase and hyaluronidase, which makes it useful for cosmetic applications [16]. However, there are no studies, to our knowledge, investigating the ability of microalgae EPS to modulate collagen production by normal human dermal fibroblasts at different ages. Moreover, the mechanism of action of these EPS still remains unclear but several evidences seem to indicate that their biological activities depend on many structural characteristics, such as sulfate content, sulfation pattern, high level of branching, uronic acid content, or reduced molecular weight [17,18].

To assess the structure–function relationships of EPS, there is consequently a compelling need to develop depolymerization methods. The most popular involve the use of enzymes, chemicals, and/or physical treatment. Enzymatic methods mainly require lyases or, to a lesser extent, hydrolases. They are simple and very specific but limited by the availability of active enzymes, enzyme thermostability, cost, and the necessity to previously have a good knowledge of the structural characteristics of the polysaccharide, such as the polysaccharidic sequence (or some repeated sequences), the regio and/or stereo-specificity of linkages between monosaccharide moieties, etc. [19]. Physical methods like the use of microwaves or ultrasounds, which was already used to depolymerize *P. cruentum* EPS [20], allow to efficiently reduce the molecular weight of polysaccharides, but their transposition at the industrial scale remains difficult and of high cost. Chemical techniques, generally realized with strong mineral acids, are frequently used to depolymerize polysaccharides but require purification steps to eliminate acid residues and the high content of produced salts [21]. One way to depolymerize completely unknown polysaccharides, excluding the use of enzymes, and avoiding the major drawbacks related to physical and/or chemical treatments, consists in using solid acid-catalyzed hydrolysis involving the use of an acidic cation-exchange resin, which enables to recover and regenerate the solid catalyst, and prevent any formation of salts during the depolymerization step, avoiding a purification step and thus reducing the cost of the process. This method has already been used by our team to successively depolymerize polysaccharides from marine macroalgae and improve their pro-collagen activity [22], but was never applied to microalgae EPS.

In this context, we stated the hypothesis that microalgae EPS may be pro-collagen stimulants and consequently potential anti-skin aging compounds for the cosmeceutical field, depending on their structural features. To bring some answers to this hypothesis, we developed in the present study a depolymerization procedure using a strong acidic cation-exchange resin, integrated in a batch or continuous reactor, in order to produce low molecular weight EPS from various microalgae, including *P. cruentum*. The influence of some structural features of the various EPS forms obtained were then assessed on the collagen production capacities of two different cell lines of normal human dermal fibroblasts. This study was conducted as part of the French Polysalgue project funded by the French National Research Agency (ANR), which aimed at identifying new strains of microalgae and cyanobacteria for the production of high value EPS. In this work, One Hundred Sixty-six original strains including One Hundred and Fifty microalgae and Sixteen cyanobacteria from diverse phylogenetic groups collected and distributed by the Roscoff Culture Collection (RCC, France; http://roscoff-culture-collection.org/) were screened for the production of EPS [23]. Forty-five positive strains were selected based on their phylogenic diversity, their promising phenotype, their belonging to species not previously described as EPS producers, and finally their ability to produce a minimum of 0.05 g.L^−1^ of EPS after 30 days of culture, under chosen culture conditions. Among these Forty-five positive strains, Seventeen new monosaccharide compositions were highlighted, and among them, Six final strains considered as the best EPS producers at a further potential industrial scale-up were chosen, taking into account that they were both positively responding to all previous criteria and original as EPS producers. These six final strains, *Chrysotila dentata* (CCAP 918/3), *Pavlova* sp. (CCAP 940/5), *Diacronema ennorea* (CCAP 914/3), *Glossomastix* sp. (CCAP 2912/1), *Phaeodactylum tricornutum* (CCAP 1055/17), and *Synechococcus* sp. (CCAP 1479/23, together with *Porphyridium cruentum*, used as a well-known model, were thus produced within the 1–3 g scale to conduct the present study, aiming at investigating both the potential of microalgae to prevent and/or reduce signs of skin aging and the structure–function relationship of such biological activity.

## 2. Materials and methods

### 2.1. Materials

*Porphyridium cruentum* EPS was provided by AlgoSource, a French company specialized in the production of ingredients and bioproducts from microalgae. EPS from *Chrysotila dentata* (CCAP 918/3), *Pavlova* sp. (CCAP 940/5), *Diacronema ennorea* (CCAP 914/3), *Glossomastix* sp. (CCAP 2912/1), *Phaeodactylum tricornutum* (CCAP 1055/17), and *Synechococcus* sp. (CCAP 1479/23) were produced and isolated according to the procedures developed and described by Gaignard et al. (2019) [23], using microalgae strains provided by the biological marine station of Roscoff (France).

All chemicals and reagents were purchased from Merck (Darmstadt, Germany). Fetal bovine serum, penicillin – streptomycin, trypsin – EDTA, and Eagle’s Minimum Essential Medium (EMEM) were purchased from Eurobio Ingen (Les Ulis, France). Trypsin was purchased from PAN Biotech (Germany).

Two cell lines of normal human dermal fibroblasts (NHDF) were obtained from ATCC Cell (Manassas, VA, USA): CCD-1059Sk (ATCC® CRL-2072^TM^, lot number 62,062,292), and CCD-1090Sk (ATCC® CRL-2106^TM^, lot number 204,756). They were derived from the skin of 20- and 46-year-old women, respectively, according to the provider’s information.

### 2.2. Depolymerization methods

#### 2.2.1 High-pressure pre-treatment

The first step of the depolymerization methods consisted in a high pressure pre-treatment aiming at reducing EPS viscosity in aqueous solution and initiating their depolymerization. This step was carried out using a TS Haiva series 2.2-kW high pressure grinder, from Constant Systems Ltd. (United Kingdom), usually designed for cell lysis. Basically, EPS solution at 20 mg/ml was placed in the grinder at a pressure of 2.7 kbar, treated during one run and transferred in a storage tank. Then, 10 ml of deionized water was injected in the grinder to wash it and thus counteract the loss of EPS material probably occurring during the treatment. These two steps were repeated four times and then washing waters and EPS solutions were assembled and freeze-dried at −80°C, using a Heto PowerDry OL 6000 lyophilization system (Thermo Electron Co., France). The obtained dried EPS was so-called high pressure pre-treated (HP-PT) form.

#### 2.2.2. Depolymerization procedure by solid acid-catalyzed hydrolysis

Two depolymerization methods were conducted by using solid acid-catalyzed hydrolysis, involving a strong acidic cation-exchange resin (Amberlyst^TM^ 15 dry; 4.7 meq H^+^/g_dry material_), integrated in a batch or recycle fixed-bed reactor.

##### 2.2.2.1 Batch system

First, 25 ml of HP-PT EPS solution at 2 mg/ml was placed in a 50 ml bottle already containing 10 g of Amberlyst^TM^ 15 dry resin previously hydrated and washed with ultrapure water. This batch system was then hermetically closed and placed in an LSE cabinet-style shaking incubator (Corning, NY, USA), maintained at 80°C under orbital shaking at 120 rpm. Then, 500 µl aliquots were collected at t_0_ and after 24 h, 48 h, and 72 h of depolymerization, frozen in an ice bath and centrifuged at 10,000 g for 5 min at room temperature. The supernatant was then neutralized at pH 7.0 by adding 1 M NaOH and freeze-dried at −80°C, using a Heto PowerDry OL 6000 lyophilization system (Thermo Electron Co., France).

##### 2.2.2.2 Recycle fixed-bed system

The recycle fixed-bed system involved a feeding tank consisting in a 50 ml bottle filled with 30 ml of 2 mg/ml HP-PT EPS solution and placed under stirring at 120 rpm, and a peristaltic pump used to allow the circulation of the EPS solution through a vertically placed glass column of 15 cm length, 1 cm inner diameter and 15 ml inner volume, packed with 10 g of Amberlyst^TM^ 15 dry resin previously hydrated and washed with ultrapure water. The EPS solution entered the column at the top and was then conducted back in the feeding tank when exiting the bottom of the column. This entire recycle system was hermetically closed and both the feeding tank and the column were maintained at 80°C using a hot plate and a thermostatic chamber (SalvisLab, Switzerland), respectively. The flow rate was set at 10 ml/min. 500 µl aliquots were collected at t_0_ and after 24 h, 48 h, and 72 h of depolymerization, frozen in an ice bath and centrifuged at 10,000 g for 5 min at room temperature. The supernatant was then neutralized at pH 7.0 by adding 1 M NaOH and freeze-dried at −80°C, using a Heto PowerDry OL 6000 lyophilization system (Termo Electron Co., France).

### 2.3. High-performance liquid size-exclusion chromatography (HPL-SEC)

The time-courses of the depolymerization reactions were followed by HPL-SEC using an Agilent Technologies 1260 Infinity HPLC/RID system involving two successive size-exclusion chromatography columns of 30 cm in size: TSK-G5000PW and TSK-G4000PW (Tosoh Bioscience GmbH, Germany). Samples were dissolved in the eluent (0.1 M sodium nitrate) at a final concentration of 20 g/l, and 20 µl was injected for analysis and eluted at 0.8 ml/min flow rate, at room temperature. Products were detected and quantified by differential refractometry using the HP ChemStation software in offline mode for processing. To estimate the M_w_ value and the percentage of oligo- and poly-saccharides of less than 10 kDa (% MW < 10 kDa), a calibration curve involving pullulan standards ranging between 0.342 and 806 kDa was used.

The absolute determination of averaged molecular weight and molecular weight distribution was performed by using an HPL-SEC system equipped with a triple detector composed of a multi-angle laser light scattering (MALS) module (Down HELEOS II, Wyatt Technology, CA, USA), a viscosity detector (VD) (Viscostar II, Wyatt Technology, CA, USA), and a differential refractive index (DRI) detector (RID 10 A, Shimadzu, Japan). Samples were dissolved in the eluent (0.1 M LiNO_3_) at a final concentration of 20 g/l, and 100 µl was injected for analysis and eluted at 0.5 ml/min flow rate through two columns in series: OHPAK SB 804 and 806 HQ (Shodex, Japan).

Weight-averaged molecular weight (M_w_), number-averaged molecular weight (M_n_), and polydispersity index (I) were calculated as follows:

(1) $$M_{n}=\left(\Sigma \;N_{i}xM_{i}\right)/\Sigma N_{i}$$

(2) $$M_{w}=\left(\Sigma \;N_{i}x{M_{i}}^{2}\right)/\left(\Sigma \;N_{i}xM_{i}\right)$$

(3) $$I\;=\;M_{w}/M_{n}$$

where N_i_ was the number of moles of polymer species and M_i_ was the molecular weight of polymer species.

### 2.4. Determination of sulfate group and uronic acid contents

Two methods were used to assess the sulfate group content of EPS. Sulfate group content of the native forms of EPS was determined by using the turbidimetric method, as described by Dodgson and Price [24]. Due to the high amount of sample material required to apply the turbidimetric method, it was not possible to use it for the depolymerized forms of EPS. For this reason, their sulfate content was estimated using 3-amino-7-(dimethylamino)phenothizin-5-ium chloride (Azure A), which binds to the sulfate groups in a polysaccharide chain [25,26], using dextran sulfate (17% sulfur) as a standard.

For all EPS forms, the uronic acid content was determined using the colorimetric method developed by Blumenkrantz and Asboe-Hansen [27] and modified by Filisetti-Cozzi and Carpita [28].

Due to numerous constraints of the project, sulfate group and/or uronic acid contents of some EPS forms could not be determined, due to either lack of material or difficulties to produce some EPS and their depolymerized forms at the time where analyses were carried out. These untested EPS forms were then specified as ‘not determined’ (nd).

### 2.5. Human matrix metalloproteinase-1 inhibition

Human matrix metalloproteinase-1 (MMP-1) inhibition assays were carried out in a 50 nM tricin buffer solution at pH 7.5, also composed of 400 nM NaCl, 10 mM CaCl_2_ and 0.1% (m/v) bovine serum albumin (BSA; added to prevent thermal denaturation of MMP-1). 20 μl of enzymatic solution (5 nM human MMP-1 in buffer solution), 20 μl of sample or positive control (100 μg/ml EPS or piroxicam in buffer solution), and 47.5 µl of buffer solution were successively added in microplate wells prior to incubation at 25°C for 10 min. Then, 12.5 μl of substrate solution (10 µM Mca-Lys-Pro-Leu-Gly-Leu-Dpa-Ala-Arg-NH_2_ solution [Mca=(7-methoxycoumarin-4-yl)-acetyl; Dpa=N-3-(2,4-dinitrophenyl)-L-α-β-diaminopropionyl]) was added and the fluorescence emitted at λ_emission_ = 390 nm (λ_excitation_ = 330 nm) was monitored using a Fluostar Omega microplate reader (BMG LABTECH, Germany) for 1 h at 25°C. A negative control corresponding to the enzymatic reaction without any inhibitor was also carried out under the same conditions. After linear regression of the linear part of the obtained curves, the initial velocities (v_i_) corresponding to the linear regression slopes were obtained, allowing to determine the percentage of human MMP-1 inhibition, using the following equation:

(4) $$Human-MMP1inhibition\left(\%\right)=100-\left(\frac{{v_{i}}_{sample}}{{v_{i}}_{negativecontrol}}\right)\times 100$$

### 2.6. Cell culture

Cells were cultured in EMEM supplemented with 10% (v/v) fetal bovine serum and 1% (v/v) antibiotic solution (10,000 U/ml penicillin, 10 mg/ml streptomycin), used as the complete culture medium. They were grown in 75 cm^2^ surface ventilated culture flasks (BD Biosciences, Franklin Lakes, NJ, USA), placed in a temperature-controlled humidified incubator with 5% CO_2_ at 37°C and subcultured by trypsinization (0.05% (*w/v*) trypsin). The culture medium was changed every two or three days. Cells were used between the third and ninth passages for the experiments.

### 2.7. Cell viability

The MTT (3-(4,5-dimethylthiazol-2-yl)-2,5-diphenyltetrazolium bromide) assay was used according to the method described by Mosmann [29]. Briefly, cells were seeded in 96-well Falcon microplates (BD Biosciences, Franklin Lakes, NJ, USA) at 5 × 10^4^ cells/ml in 100 μl of complete culture medium and incubated for 24 h. The medium was then removed and 100 μl of sample prepared in EMEM containing 1% (v/v) antibiotic solution or negative control (EMEM containing 1% (v/v) antibiotic solution without sample) was added in the wells. After 48 h, 25 µl of MTT (5% (*w*/*v*) in PBS) was added in each well and the microplates were incubated again for 4 h. The medium was removed and 200 μl of dimethyl sulfoxide was added in each well. The microplates were then incubated for 10 min prior to absorbance reading at 550 nm using a Fluostar Omega microplate reader (BMG LABTECH, Germany).

Cell viability was obtained as follows:

(5) $$Cellviability\left(\%\right)=\frac{{A_{550nm}}_{sample}-{A_{550nm}}_{negativecontrol}}{{A_{550nm}}_{negativecontrol}}\times 100$$

### 2.8. Cell necrosis

The lactate dehydrogenase (LDH) test was used to assess cell necrosis. This method is based on the loss of the cell membrane integrity occurring during cell death, which leads to the release of cytoplasmic enzymes such as lactate dehydrogenase (LDH) into the extracellular medium. It measures the activity of LDH released by damaged cells in the cell supernatant, good marker of cell death [30].

LDH release was measured with a commercially available LDH assay kit (Cytotoxicity Detection Kit, Roche, France), following the supplier’s instructions. Briefly, the preparation of microplates was identical to that used for cell viability assessment, except that a final volume of 200 µl and Triton 1X as a positive control were used. After 48 h of exposure, microplates were centrifuged at 250 g for 10 min at room temperature. 100 µl of cell supernatant was then added in a new microplate well, prior to adding 100 µl of mixture reagent and incubating at 22°C for 30 min in the dark. The absorbance of the red colored formazan salt produced was directly measured at 492 nm using a Fluostar Omega microplate reader (BMG LABTECH, Germany).

Cell necrosis was obtained as follows:

(6) $$Cellnecrosis\left(\%\right)=\frac{{A_{492nm}}_{sample}-{A_{492nm}}_{negativecontrol}}{{A_{492nm}}_{positivecontrol}-{A_{492nm}}_{negativecontrol}}\times 100$$

### 2.9. Quantification of collagen production

The Sirius Red coloration was used for determination of cell collagen production [31]. 500 µl of NHDF suspension in complete culture medium was seeded in 24-well microplates (5 × 10^4^ cells/well) and incubated for 24 h. The medium was then substituted by 500 µl of 100 µg/ml sample or a negative or positive control in EMEM containing 1% (*v*/*v*) antibiotic solution, and the microplates were incubated again for 48 h. The medium was removed and cells were washed with PBS and then fixed for 1 h with 1 ml of Bouin’s solution at room temperature. After fixation, the Bouin’s solution was removed and cells were washed twice with ultrapure water and stained with 1 ml of Sirius Red solution (0.5 g of Sirius Red in 500 ml of saturated picric acid aqueous solution) for 1 h, under stirring at room temperature. Cells were then washed successively with ultrapure water (twice) and 0.01 M HCl to remove unbound dye. The bound dye was finally solubilized in 250 μl of 0.1 M NaOH for 1 h under stirring and absorbance was read at 550 nm using a Fluostar Omega microplate reader (BMG LABTECH, Germany).

Results were expressed as the mean relative percentage of collagen production compared to the negative control, using the following equations:

(7) $$Collagenproductionperwell\left(\%\right)=\frac{{\left[collagen\right]}_{sample}}{{\left[collagen\right]}_{negativecontrol}}\times 100$$

(8) $$Collagenproductionpercell\left(\%\right)=\frac{collagenproductionperwell\left(\%\right)}{cellviability\left(\%\right)}\times 100$$

### 2.10. Statistical analysis

All data are presented as means ± standard deviations of at least triplicates. The student’s t-test (independent, two-sided) was used to determine significant differences between experimental and control samples, using Origin 6.0 software (OriginLab, Northampton, MA, USA).

Principal component (PCA) and Pearson analyses were conducted using Sigma Plot 14.0 software (Systat Software Inc., San Jose, CA, USA) to compare the major differences, mostly structural, between the four forms of the seven EPS of the study and their effect on their pro-collagen activity. EPS forms were thus used as individuals while M_w_, M_n_, I, % MW < 10 kDa, sulfate group and uronic acid contents, and cell viability were used as variables in the PCA, together with either inhibition of MMP-1 or collagen production per cell.

## 3. Results and discussion

### 3.1. Production and depolymerization of EPS from microalgae

#### 3.1.1. Production and characterization of EPS

**Figure 1.**
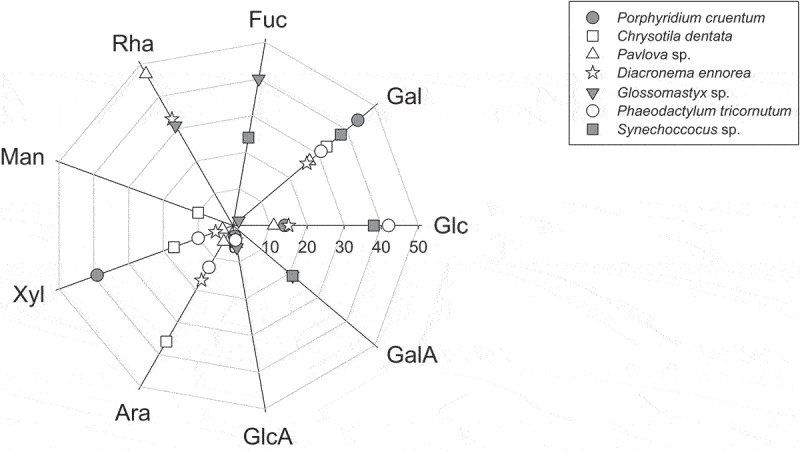
Monosaccharide compositions of EPS from *P. cruentum*, *C. dentata*, *Pavlova* sp., *D. ennorea*, *Glossomastix* sp., *P. tricornutum* and *Synechococcus* sp., as determined by GC/MS analysis by Gaignard et al. (2019) [23]: Man: mannose; Rha: rhamnose; Fuc: fucose; Gal: galactose; Glc: glucose; GalA: galacturonic acid; GlcA: glucuronic acid; Ara: arabinose; Xyl: xylose.

Seven EPS were produced and isolated from different species of microalgae, according to the procedure developed and described by Gaignard et al. [23]: *Porphyridium cruentum*, *Chrysotila dentata* (CCAP 918/3), *Pavlova* sp. (CCAP 940/5), *Diacronema ennorea* (CCAP 914/3), *Glossomastix* sp. (CCAP 2912/1), *Phaeodactylum tricornutum* (CCAP 1055/17), and *Synechococcus* sp. (CCAP 1479/23). The monosaccharide composition of these seven EPS was characterized by GC/MS analysis, confirming the well-known composition of *P. cruentum* EPS, which is mainly composed of galactose (44%), xylose (39%), and glucose (14%) (Figure 1). It also revealed that EPS from *C. dentata*, *Pavlova* sp., *D. ennorea*, *P. tricornutum*, and *Synechococcus* sp. are mostly composed of galactose (within the range 26–38%) and arabinose/xylose (36%/17%), rhamnose/glucose (47%/11%), rhamnose/arabinose (33%/17%), glucose/arabinose (42%/13%), and glucose/fucose (38%/24%), respectively, while *Glossomastix* sp. EPS is mostly composed of fucose/rhamnose/galacturonic acid (40%/31%/21%). On the other hand, all EPS except the one from *Synechococcus* sp. were anionic polysaccharides, all including low proportions of glucuronic acid (within the range 4–6%) as *Glossomastix* sp. EPS also exhibited a high proportion of galacturonic acid. The monosaccharide composition of *P. cruentum* EPS was in perfect accordance with the literature [32,33], indicating that a reasonable confidence could be credited to the monosaccharide compositions of the other unknown EPS, which were in accordance with the high diversity found in external polysaccharides from microalgae as well [13,34,35].

These EPS were very high size polysaccharides (Table 1), the smallers, from *P. cruentum* and *Synechococcus* sp., exhibiting M_w_ of 1820 and 3500 kDa, respectively, as all the others exceeded 5000 kDa, reaching 9500 kDa for the heaviest, produced by *C. dentata* CCAP 918/3. Unfortunately, the molecular weight of *Glossomastyx* sp. EPS could not be determined, as it could not be properly separated under the conditions used for HPL-SEC/MALS-VD-DRI analysis, even after optimization, most likely due to the formation of aggregates. Regarding the native form of *P. cruentum* EPS, we obtained very similar results to the works of Geresh et al. regarding *P. cruentum* EPS [13], and Gargoush et al. regarding *P. marinum* EPS [36].

**Table 1.** Structural characterization of the different forms of EPS: native and depolymerized after high pressure pre-treatment (HP-PT) or solid acid-catalyzed hydrolysis in batch (B-dep) or recycle fixed-bed (RFB-dep) reactor systems.

EPS	EPS source	Form	M_w_ (kDa)	M_n_ (kDa)	I	% MW < 10 kDa	Sulfate group content (%)	Uronic acid content (%)
Pc	*Porphyridium cruentum*	Native	1820	1270	1.4	48	12.3^b^	7.3
		HP-PT	1000	400	2.5	28	9.8^c^	nd^a^
		B-dep	19	6.4	3.0	65	0.2^c^	7.3
		RFB-dep	14	4.2	3.3	83	0.3^c^	6.4
Cd	*Chrysotila dentata* (CCAP 918/3)	Native	9500	4320	2.2	68	9.7^b^	7
		HP-PT	2100	500	4.2	40	10.9^c^	nd^a^
		B-dep	30	2.7	11.1	84	0.2^c^	6
		RFB-dep	15	2.3	6.5	89	0.1^c^	6
P	*Pavlova* sp. (CCAP 940/5)	Native	5600	4000	1.4	48	10.6^b^	4
		HP-PT	nd^a^	400	nd^a^	38	nd^a^	nd^a^
		B-dep	110	82	1.4	86	1.2^c^	5
		RFB-dep	100	60	1.7	96	0.7^c^	5
De	*Diacronema ennorea* (CCAP 914/3)	Native	6100	3000	2.0	37	10.6^b^	8
		HP-PT	1500	300	5.0	39	nd^a^	nd^a^
		B-dep	730	57	12.8	81	1.1^c^	4
		RFB-dep	1200	40	30.0	87	1.4^c^	5
G	*Glossomastix* sp. (CCAP 2912/1)	Native	nd^a^	nd^a^	nd^a^	66	13.7^b^	26
		HP-PT	1250	600	2.1	21	nd^a^	nd^a^
		B-dep	140	15	9.3	89	4.7^c^	24
		RFB-dep	290	17	17.1	80	4.4^c^	24
Pt	*Phaeodactylum tricornutum* (CCAP 1055/17)	Native	5000	1500	3.3	57	13.9^b^	4
		HP-PT	3200	800	4.0	30	13.8^c^	nd^a^
		B-dep	2500	240	10.4	65	0.9^c^	4
		RFB-dep	2000	140	14.3	81	0.7^c^	5
S	*Synechococcus* sp. (CCAP 1479/23)	Native	3500	3100	1.1	53	18.7^b^	3
		HP-PT	2230	420	5.3	32	22^c^	nd^a^
		B-dep	74	13	5.7	97	0.3^c^	3
		RFB-dep	95	7	13.6	99	0.3^c^	3

^*a*^*Not determined*.

^*b*^*Determined by turbidimetric assay*.

^*c*^*Determined by Azure A assay*.

#### 3.1.2. Determination of the optimal depolymerization conditions

EPS in their native form were very hardly depolymerized by direct solid acid-catalyzed hydrolysis. Indeed, it took too much time to efficiently reduce their size, up to 4–5 days (data not shown), most likely due to their very high molecular weight and steric hindrance phenomena, preventing them to diffuse into the resin micro-environment. For this reason, high pressure homogenization was first carried out as a pre-treatment step, to start reducing both the molecular weight and viscosity in solution of the native EPS. EPS in solution were placed in a grinder at the maximal pressure allowed of 2.7 kbar, and submitted to five successive cycles, which are necessary conditions to reduce the molecular weight of algal polysaccharides in general by about 2–3, based on previous studies performed by one of the partner teams of the project [19,36].

EPS forms obtained after this first depolymerization step, named HP-PT EPS, reflected the expected high decrease in their native molecular weight, by about 46–75% (Table 1).

**Figure 2.**
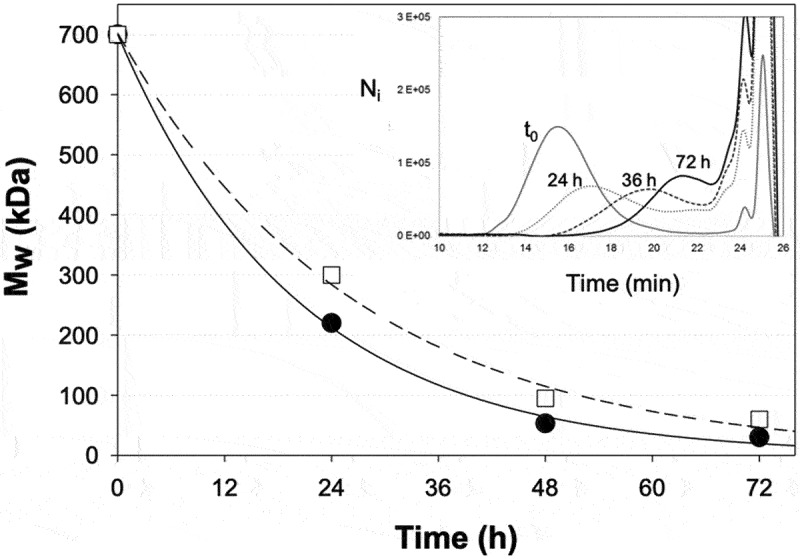
Time-course evolution of the weight-averaged molecular weight (M_w_) of *P. cruentum* EPS during its depolymerization in batch (—) or recycle fixed-bed (---) reactor systems, and example of HPL-SEC time course monitoring of *P. cruentum* EPS depolymerization carried out in the recycle fixed-bed reactor.

Starting from these HP-PT forms of EPS, we then developed a depolymerization procedure by solid acid-catalyzed hydrolysis, involving a strong acidic cation-exchange resin integrated in a batch or recycle fixed-bed reactor, in order to produce low molecular weight EPS. To determine the best depolymerization conditions, only the EPS from *P. cruentum* was tested, monitoring the time-course of the formation of low molecular weight polysaccharides, around 20 kDa, by HPL-SEC chromatography (Figure 2).

Both depolymerization systems successively reduced the molecular weight of *P. cruentum* EPS, with a better efficiency for the batch system. Indeed, the molecular weight obtained after 48 h of reaction with this system was estimated to approximately 3.5% of its initial value, against about 6% with the recycle fixed-bed system. To obtain similar values of M_w_, it was necessary to wait for 24 h more, giving drastic decreases in the initial M_w_ value of *P. cruentum* EPS, close to 2% and 4% for the batch and recycle fixed-bed systems, respectively. The better efficiency of the batch reactor system to depolymerize this EPS might be due to a better distribution between EPS and the interface of the cation-exchange resin, which could be caused by a reduction of diffusion limitations under batch conditions.

Taking these results into consideration, we then decided to transpose the two methods to the depolymerization of all EPS, choosing 48 h and 72 h as ending times of reaction for the batch (B-dep EPS) and recycle fixed-bed (RFB-dep EPS) systems, respectively, and determined their values of weight-averaged and numbered-averaged molecular weight, as well as their polydispersity index (Table 1). The M_w_ of native, HP-PT, B-dep, and RFB-dep forms were determined at 1820 kDa, 1000 kDa, 19 kDa and 14 kDa, respectively, for *P. cruentum* EPS, confirming that both depolymerization methods enabled to reduce the initial molecular weight of this EPS by more than 99%. The other EPS gave very similar results, their B-dep and RFB-dep forms exhibiting M_w_ an M_n_ values ranging within 15–290 kDa (97.9–99.8% M_w_ reduction) and 2.3–82 kDa (98.5–99.9% M_n_ reduction), respectively, except for the EPS from *P. tricornutum* and *D. ennorea*, which were not fully depolymerized, reaching only 60.0–88.0% M_w_ reduction and 90.7–98.7% M_n_ reduction, respectively. These two EPS were thus very resistant to solid acid-catalyzed hydrolysis. The M_w_ value of *Pavlova* sp. HP-PT EPS form could not be determined, most likely for the same reason previously described that prevented to characterize M_w_ and M_n_ values of *Glossomastix* sp. native EPS form.

**Figure 3.**
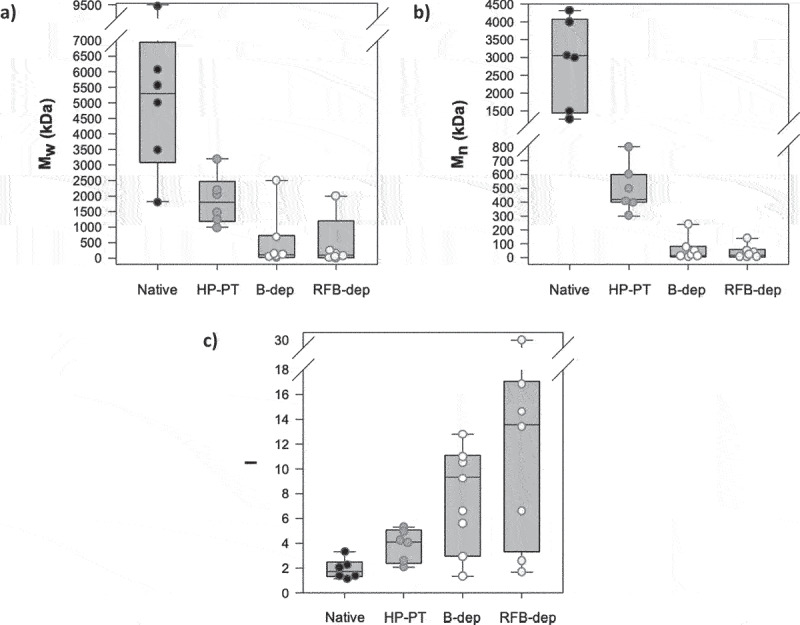
Value distributions of M_w_ (a), M_n_ (b), and I (c) for the seven microalgae EPS, under their native and depolymerized forms obtained after high pressure pre-treatment (HP-PT) and solid acid-catalyzed hydrolysis in batch (B-dep) or recycle fixed-bed (RFB-dep) reactor systems.

Overall, the better efficiency of the batch reactor system to depolymerize these EPS was confirmed, as the M_w_ distribution of B-dep EPS was narrower than with the recycle fixed-bed system (Figure 3). Interestingly, M_n_ distributions were very similar, revealing that the two systems were equivalently efficient to depolymerize the most represented polysaccharides of the EPS samples. On the other hand, the polydispersity of all EPS, varying within the range 1.1–3.3, was shown to logically strongly increase after depolymerization, again depending on the considered EPS, most likely due to the formation of a mixture of low molecular weight poly- and/or oligo-saccharides indicated by the high decrease in the M_n_ value. Logically, the polydispersity index distribution was little less extended with the batch system.

Both methods of depolymerization by solid acid-catalyzed hydrolysis preserved the uronic acid content of EPS, but drastically reduced their sulfate content. Indeed, apart from *Glossomastix* sp. EPS that lost only about 70% of its sulfate groups, all other EPS B-dep and RFB-dep forms were almost completely desulfated, exhibiting only from 0.01% to 2.4% of residual sulfate groups. This result was expected, since acid hydrolysis is well-known to cause the rupture of sulfate monoesters, especially when using a very strong acidic cation-exchange resin. Less acidic resins could be used to moderate this phenomenon and partially conserve sulfate groups, which was previously demonstrated by our team to depolymerize ulvan-type polysaccharides from *Ulva* sp. macroalgae using a two-times less acidic resin [22]. But the resistance to acid hydrolysis of some EPS, from *P. tricornutum* and *D. ennorea* in particular, constrained us to use a stronger acidic resin in this case. Only the high pressure pretreatment step seemed to preserve EPS sulfation, as we observed that sulfate group contents of EPS HP-PT forms from *P. cruentum*, *C. dentata*, *P. tricornutum*, and *Synechococcus* sp. were very close to the values of their native forms. This means that this pretreatment step was both harsh enough to initiate depolymerization of EPS and soft enough to preserve the sulfate ester bounds on the polysaccharide chains. Besides, it is noteworthy that some values were higher after HP-PT pretreatment, which is impossible. This was most likely due to the Azur A method that was used for the depolymerized forms of EPS, which is much more inaccurate than the turbidimetric method that was used to estimate the sulfate group contents of native forms only.

Nevertheless, the twenty-eight different forms of generated EPS were shown to exhibit various determined structural features, including high, intermediary, and low molecular weight (differences in M_w_, M_n_, and I), sulfate group content and uronic acid content, which allowed us to investigate both the potential of microalgae EPS as anti-aging compounds and to study the structure–function relationship of this particular biological activity. In this purpose, we focused on pro-collagen activities.

### 3.2. Human matrix metalloproteinase-1 inhibition effect of the seven microalgae EPS

The first evaluated pro-collagen activity of the seven microalgae EPS was their inhibition effect toward a key enzyme of the collagen degradation process: the interstitial collagenase, also known as matrix metalloproteinase-1 (MMP-1), which is the only collagenase capable of breaking collagen fibers into fragments. Piroxicam, a well-known inhibitor of MMP-1 (IC_50_ = 63 µM) [37], was used as a positive control.

**Figure 4.**
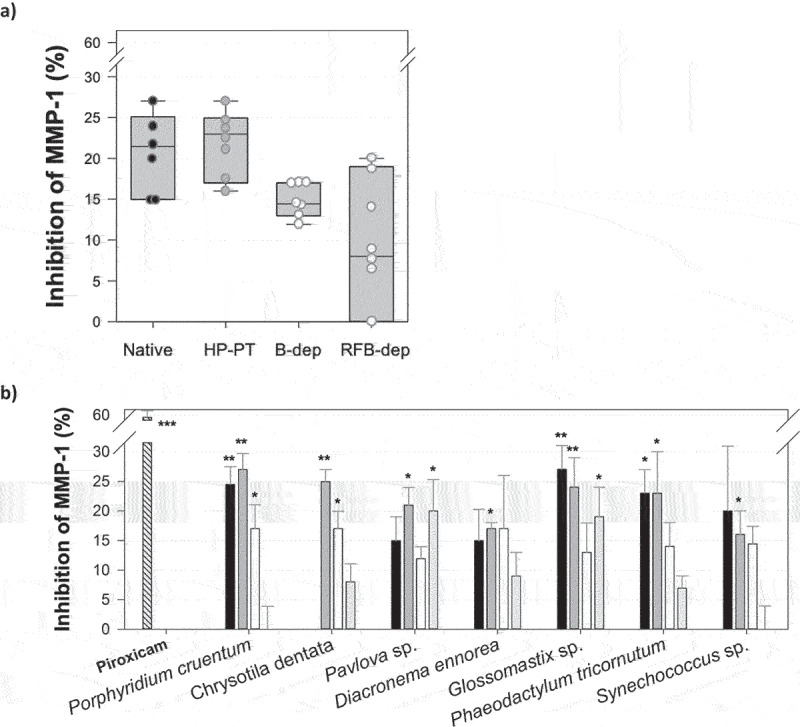
Inhibition of human MMP-1 by the seven microalgae EPS, at 100 µg/ml under their native (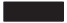) and depolymerized forms obtained after high pressure pre-treatment (HP-PT: 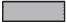) and solid acid-catalyzed hydrolysis in batch (B-dep: 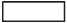) or recycle fixed-bed (RFB-dep: 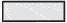) reactor systems: box plots (a) and bar charts obtained with EPS compared to piroxicam at 100 µg/ml used as a positive control (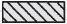) (b). Significant differences between values obtained with samples and negative control are indicated by * (*p* < 0.05), ** (*p* < 0.01), and *** (*p* < 0.001): *N* = 5.

All EPS inhibited human MMP-1, inducing a reduction of its activity ranging within 15–28% under their native and HP-PT forms (Figure 4a). More depolymerized forms (B-dep and RFB-dep) were less active, giving 20% inhibition of MMP-1 at the most. Again, B-dep forms were all very similar in terms of inhibition activity, while RFB-dep forms gave very heterogenous results. EPS from *P. cruentum* and *Glossomastyx* sp. were particularly attractive as their native and HP-PT forms significantly inhibited human MMP-1 (Figure 4b). The native and HP-PT forms of *Glossomastyx* sp. EPS were the most active of all EPS, giving about 28% inhibition at 100 µg/ml, which is only two-fold less active than piroxicam (58% inhibition at the same concentration).

### 3.3. Effect of the EPS forms on collagen production by normal human dermal fibroblasts

#### 3.3.1. Evaluation of the cytotoxicity of the EPS forms

Prior to investigating the pro-collagen activity of the different forms of the seven microalgae EPS, it was essential to determine their potential cytotoxicity. This was realized by cell viability and cell necrosis analyses using MTT and LDH assays, respectively, associated to morphological analysis by optical spectroscopy. Hyaluronic acid (HA), a glycosaminoglycan produced by fibroblasts in the dermis extracellular matrix and well-known stimulant of collagen biosynthesis by dermal fibroblasts *in vivo* or *in vitro* [5,38–40], was used as a positive control, so its cytotoxicity was evaluated as well.

Regarding cell viability (Figure S1), hyaluronic acid did not affect cell viability while the four forms of all EPS led to a decrease in cell viability of both cell models, within the ranges 14–38% and 30–58% for fibroblasts CDD-1059Sk and CDD-1090Sk, respectively. RFB-dep forms of EPS were shown to have the more drastic effect on cell viability overall. However, absolutely no cytotoxic effect was induced by these EPS, whatever their form be. Indeed, they did not have any pro-necrotic effect on both cell lines, as proved by LDH test (Figure S2): none of them had a pro-necrotic effect, except the native form of *C. dentata* EPS, which was shown to significantly increase cell necrosis of both cell lines, by 11% and 27%, respectively, compared to the negative control. RFB-dep forms of *Glossomastyx* sp. and *P. tricornutum* EPS also exhibited a very low pro-necrotic effect on CDD-1090Sk fibroblasts, reaching less than 9% and 7% cell necrosis, respectively. Moreover, only the native form of *C. dentata* EPS affected the morphology of the cells during their whole culture kinetics, as monitored by optical microscopy, which would indicate that it might have pro-apoptotic properties, but none of the other EPS forms led to such observation. Besides, all EPS were diafiltered by tangential flow filtration using a 50 kDa cutoff [23] to both concentrate and remove non chelated salts and potential remaining chemicals, which could have been responsible for potential cytotoxicity.

Altogether, these results indicate that only the native form of *C. dentata* EPS should be considered cytotoxic, suggesting that the decrease in fibroblast viability observed with the other EPS by MTT analysis was most likely due to a cytostatic effect. Hence, due to its cytotoxic property, the native form of *C. dentata* EPS was not studied in the next part of the work.

#### 3.3.2. Collagen production by fibroblasts exposed to EPS

**Figure 5.**
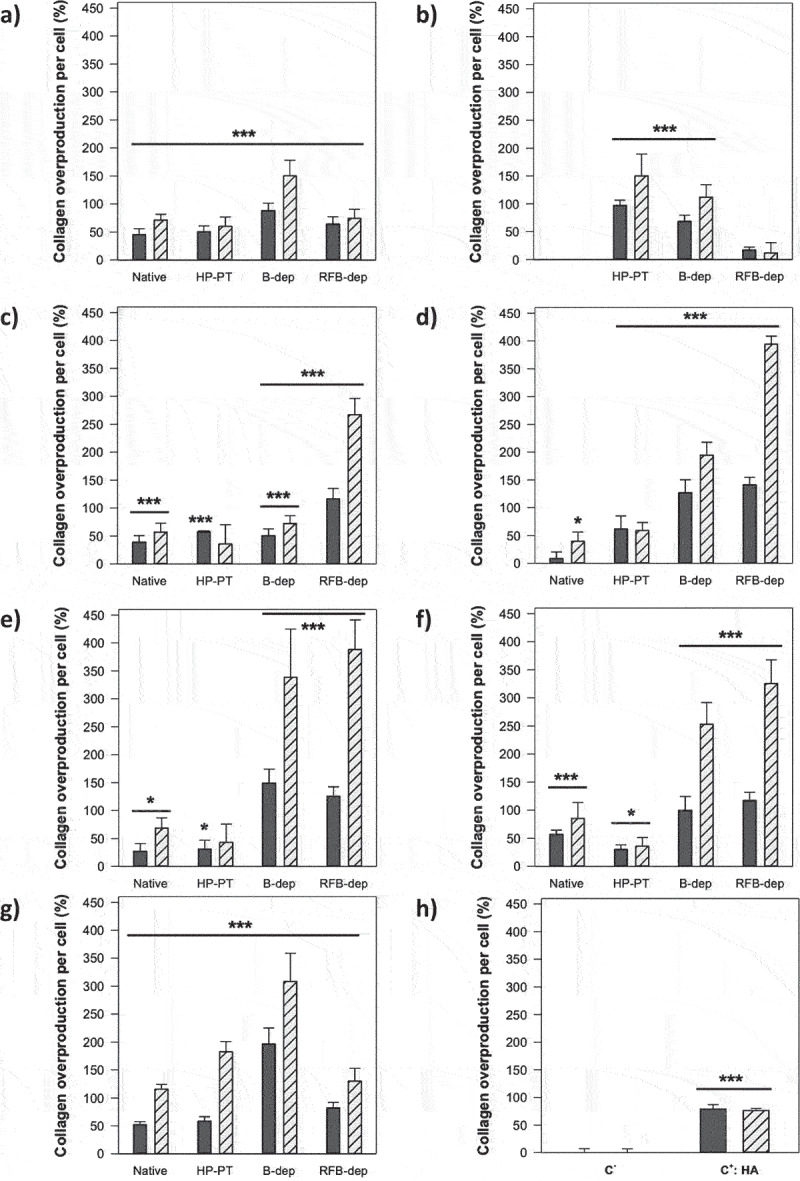
Effect of the seven microalgae EPS (a, *P. cruentum*; b, *C. dentata*; c, *Pavlova* sp.; d, *D. ennorea*; e, *Glossomastix* sp.; f, *P. tricornutum*; g, *Synechococcus* sp.) at 100 µg/ml under their native and depolymerized forms obtained after high pressure pre-treatment (HP-PT) and solid acid-catalyzed hydrolysis in batch (B-dep) or recycle fixed-bed (RFB-dep) reactor systems, on collagen production by fibroblasts CDD-1059Sk (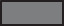) and CDD-1090Sk (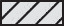) in culture. Hyaluronic acid at 1000 µg/ml (HA) was used as a positive control (h, C^+^). Results are expressed as the percentage of produced collagen compared to the negative control (C^−^). Significant differences between values obtained with samples and negative control are indicated by * (*p* < 0.05), ** (*p* < 0.01), and *** (*p* < 0.001): *n* = 9, *N* = 3.

The effect of the seven microalgae EPS under their native and three depolymerized forms was evaluated *in vitro* on the production of collagen by normal human dermal fibroblasts in culture. Both cell lines CDD-1059Sk and CDD-1090Sk were thus exposed to 100 µg/ml EPS, prior to determining the collagen production per cell, i.e. the amount of produced collagen measured by Sirius Red assay, related to the number of viable cells quantified by MTT assay. As expected, the use of 1000 µg/ml HA very similarly enhanced collagen production of both CDD-1059Sk and CDD-1090Sk cell lines, by 79% and 76%, respectively (Figure 5).

All native and HP-PT forms of EPS were also shown to stimulate collagen production, within the range 8–150%, also very similarly for both cell lines. More interestingly, most of the depolymerized forms of EPS had much higher pro-collagen activity, increasing collagen production by at least 100% for the less active and more than 300% for the most active. For example, collagen production was enhanced by about 394%, 388%, and 326%, by the RFB-Dep forms of the EPS from *D. ennorea*, *Glossomastyx* sp., and *P. tricornutum*, respectively, as the B-dep form of *Synechococcus* sp. EPS stimulated collagen production by about 308%. It is noteworthy that some differences were observed between results obtained with the two depolymerized forms, suggesting that the depolymerization method had a significant effect on the pro-collagen activity of EPS and that the two depolymerization systems most likely led to the formation of different poly- and/or oligo-saccharide structures. Some structural analyses are currently ongoing to investigate this assumption.

Moreover, this pro-collagen activity was shown overall to be significantly higher with the CDD-1090Sk cell line, which is coming from an older female donor than the CDD-1059Sk cell line. This tends to indicate that the stimulation effect of depolymerized EPS is strongly modulated by the physiological state of fibroblasts, and is of great interest to envisage further development of these compounds as anti-aging agents in the cosmeceutical field. Indeed, both depolymerized forms of EPS were active on both fibroblast cell lines, indicating that these EPS would be of interest in order to either prevent or reduce signs of skin aging.

At last, these results are in complete accordance with the negative effect of the depolymerized forms of EPS observed on the cell viability of fibroblasts, together with their lack of pro-necrotic or pro-apoptotic effect previously shown (see part 3.3.1.), which seemed to highlight a cytostatic effect of EPS on fibroblasts. With the metabolism of fibroblasts exposed to EPS being strongly reoriented toward biosynthesis of collagen, it would make sense that their growth rate is consequently negatively affected. Besides, this phenomenon was quite expected, as our team showed a very similar behavior of the same kind of dermal fibroblasts exposed to ulvans from *Ulva* sp. macroalgae [22], and this was also demonstrated by a study of Mast et al. [38], which showed that the pro-collagen effect of HA on dermal fibroblasts is associated to a decrease in cell viability involving absolutely no decrease in DNA concentration and thus no increase in cell mortality.

### 3.4. Principal component analysis

**Figure 6.**
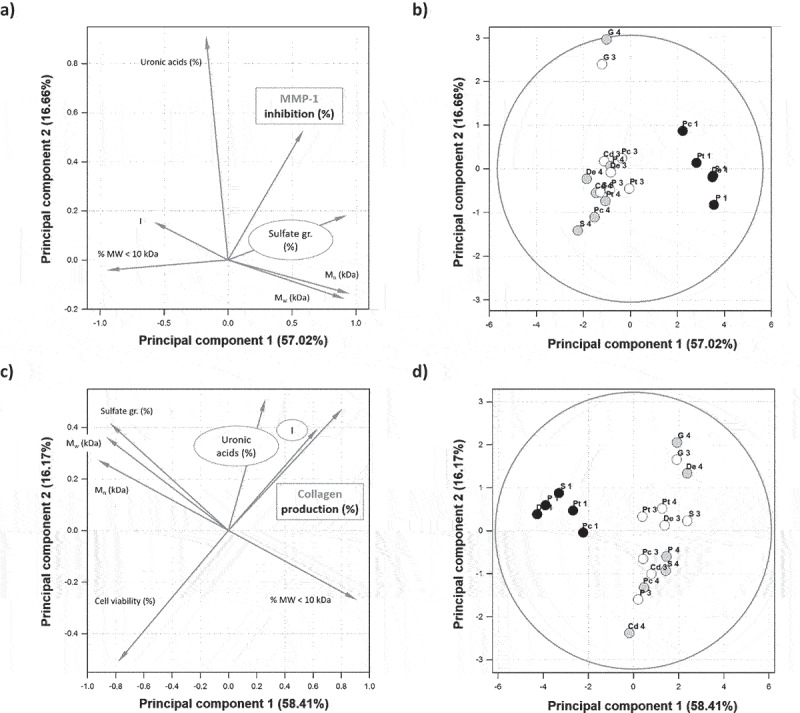
Principal component analysis (PCA) of the effect of the seven microalgae EPS under their native and depolymerized forms obtained after high pressure pre-treatment (HP-PT) and solid acid-catalyzed hydrolysis in batch (B-dep) or recycle fixed-bed (RFB-dep) reactor systems, on the inhibition of MMP-1 (a: loadings; b: scores) and the collagen production by fibroblasts CDD-1059Sk (c: loadings; d: scores): EPS from *P. cruentum* (Pc), *C. dentata* (Cd), *Pavlova* sp. (P), *D. ennorea* (De), *Glossomastix* sp. (G), *P. tricornutum* (Pt), and *Synechococcus* sp. (S), under their native (1), B-dep (3), and RFB-Dep (4) forms.

From a structural point of view, we observed several major structural differences between the four forms of the seven EPS of this study (Table 1 and Figure 3), especially in terms of molecular weight and uronic acid and sulfate groups contents. In order to better understand the structure–function relationship of the pro-collagen activity of these EPS, we thus performed a PCA, aiming at highlighting the features, mostly structural, which were mainly involved in this biological activity. Weight-averaged molecular weight (M_w_), number-average molecular weight (M_n_), proportion of polysaccharides of less than 10 kDa MW value (% MW < 10 kDa), sulfate group and uronic acid contents, and cell viability were thus used as variables evaluated on either inhibition of MMP-1 or collagen production per cell. As PCA strictly requires numeric values for all studied variables, HP-PT forms of EPS were not considered in this PCA, due to their uronic acid and sulfate content missing values. In both cases, a clear difference was logically noticed between native and depolymerized forms of EPS (Figure 6b,d).

The first PCA led to the obtention of two components explaining 73.68% of variance of the data set (Figure 6a). It especially showed that the inhibition of MMP-1 by EPS might be strongly correlated to their sulfate group content. Indeed, these two variables were found really close to each other in the same quadrant of the PCA loading plot. On the contrary, the proportion of low molecular weight polysaccharides, less than 10 kDa, is absolutely not correlated to this inhibition effect, and might actually be even inversely correlated, which is confirmed by the fact that both M_w_ and M_n_ variables were not found in the same quadrant than MMP-1 inhibition but still relatively close. Besides, the PCA also highlighted that MMP-1 inhibition is likely not correlated to EPS uronic acid content. All these conclusions were confirmed by calculating Pearson correlation coefficients (Table 2), which showed that the only structural features exhibiting a *p*-value inferior to 0.05 and thus correlated to MMP-1 inhibition were % MW < 10 kDa (inversely correlated) and sulfate group content (directly and strongly correlated). Synthetic inhibitors of MMP-1 usually exhibit hydroxamate, carboxylate, sulfhydryl, or thiol groups, which often act by competitively interacting with Zn^2+^ ions in the active site of the enzyme, mainly by hydrogen binding [37]. This could explain why sulfate groups, which also could be involved in such interactions, seem so important for MMP-1 inhibition, but this is not in accordance with the absence of correlation with EPS uronic acid content highlighted by the PCA. This would also explain why the depolymerization of EPS led to the loss of MMP-1 inhibition activity, since it drastically reduced their sulfate group content. In accordance with our results, Shirzad et al. also showed that some bacterial EPS could inhibit a bacterial collagenase derived from *Clostridium histolyticum* [41], but these authors did not highlight any structural or mechanistic feature related to this biological activity, preventing any further discussion of this aspect.

**Table 2.** Correlation between inhibition of MMP-1 or collagen production by fibroblasts CDD-1059Sk and CDD-1090Sk in culture and microalgae EPS structural features or cell viability.

Biological activity	M_w_ (kDa)	M_n_ (kDa)	I	% MW < 10 kDa	Sulfate group content (%)	Uronic acid content (%)	Cell viability (%)
Inhibition of MMP-1 (%)	PCA correlation coefficient						
	0.322	0.338	−0.324	−0.497	0.557	0.122	-
	Pearson correlation coefficient (*p-*value)						
	0.322 *(0.179)*	0.338 *(0.157)*	−0.324 *(0.176)*	−0.496 *(0.026)*	0.618 *(0.00367)*	0.326 *(0.161)*	-
Collagen production per cell (%)	PCA correlation coefficient						
	−0.479	−0.549	0.517	0.598	−0.444	0.252	−0.934
	Pearson correlation coefficient (*p-*value)						
	−0.479 *(0.0378)*	−0.549 *(0.0149)*	0.517 *(0.0235)*	0.602 *(0.00496)*	−0.494 *(0.0267)*	0.0436 *(0.855)*	−0.939 *(0.898×10*^*-9*^)

Regarding the second PCA, which focused on the direct pro-collagen activity of EPS, it gave two components explaining 74.58% of variance of the data set (Figure 6c). More interestingly, it showed that collagen production per cell might be mostly correlated to the polydispersity and uronic acid content of EPS, and not to their other features, except % MW < 10 kDa, whose loading vector was not found in the same quadrant than collagen production per cell but relatively close. This was quite unexpected because it does not bring clear explanation of why depolymerization of native EPS led to a significant increase in the pro-collagen activity. Very similar results were obtained with the data set corresponding to collagen production by fibroblasts CDD-1090Sk (Figure S3). Pearson correlation analysis was essential in this case, as it showed no direct correlation of collagen production per cell with uronic acid content of EPS (*p*-value >0.05) but a very clear direct correlation with their polydispersity index and even more with % MW < 10 kDa (Pearson correlation coefficient: 0.602; *p*-value <0.005). Altogether, PCA and Pearson analyses indicate that low molecular weight forms of microalgae EPS are essential for their pro-collagen activity, while uronic acid content is very important but only if the size of EPS is reduced in the first place. One likely explaining hypothesis might be that the production of low molecular weight poly- and/or oligo-saccharides during depolymerization allows some hidden areas of the polysaccharide backbones to be released or at least exposed at the surface of the polysaccharide and then free to be involved in the mechanism(s) leading to collagen production activation. This highly speculative assumption obviously needs to be investigated by structural analysis of the depolymerized polysaccharides, which will be considered soon. However, uronic acid content is an important feature of anionic polysaccharides that has already been shown to be essential for many of their biological activities, such as antioxidant properties or inhibition of α-glucosidase [18,42]. Besides, we used hyaluronic acid as a positive control for its well-known pro-collagen activity, which is also in accordance with the results of these analyses. Hyaluronic acid is indeed composed of a very high content of glucuronic acid (46%). Nevertheless, collagen production by fibroblasts exposed to 1000 µg/ml hyaluronic acid was not enhanced at the same level than with most of the EPS forms of our study, which indicates that other structural characteristics are involved in this pro-collagen activity. One of these could have been sulfate content, which is a structural feature that hyaluronic acid does not exhibit, but both PCA and Pearson analyses invalided this possibility.

Furthermore, PCA and Pearson analyses demonstrated that cell viability is strictly inversely correlated to collagen production per cell (Pearson correlation coefficient: −0.939; *p*-value <0.001), which was expected as it confirms the conclusion we previously proposed to explain that no cytotoxicity in terms of pro-necrotic or pro-apoptotic effect toward fibroblasts was observed with any of the EPS forms involved in the PCA.

## 4. Conclusion

We developed in this study a new depolymerization method, which combined high pressure pre-treatment and solid acid-catalyzed hydrolysis, to efficiently reduce the molecular weight of seven EPS from microalgae, among which six were new and original. The applied procedure was developed in a batch or recycle fixed-bed reactor, which are methods that could be scaled-up for potential industrial application. The four different EPS forms obtained (native, high pressure pre-treated, depolymerized in a batch, or recycle fixed-bed reactor) were shown to inhibit matrix metalloproteinase-1 (27% inhibition induced by the native most active forms). More interestingly, they were also able to promote collagen production by two cell lines of human dermal fibroblasts, reaching 300–400% stimulation for the most active: *D. ennorea*, *Glossomastix* sp., and *P. tricornutum* EPS depolymerized forms obtained in the recycle fixed-bed reactor and *Synechococcus* sp. EPS depolymerized form obtained in the batch reactor. Starting from the structural characterization of these EPS that was partially achieved, we performed principal component and Pearson statistical analyses to highlight the features mainly involved in these biological activities. On the one hand, MMP-1 inhibition by microalgae EPS was shown to be strongly correlated to their sulfate group content. On the other, collagen production by fibroblasts was mostly correlated to their proportion of low molecular weight polysaccharides (<10 kDa), while their uronic acid content was shown essential but only if EPS size was previously reduced.

Altogether, these results demonstrate that four of the six new EPS that we isolated, from *D. ennorea*, *Glossomastyx* sp., *P. tricornutum*, and *Synechococcus* sp., are strong pro-collagen stimulants and consequently have a high potential to envisage further development as anti-skin aging compounds for the dermo-cosmeceutical field. They also tend to prove that pro-collagen activity of EPS depends on a few key structural parameters, which could be considered for the research of new active polysaccharides in the field.

## Supplementary Material

Supplemental Material

## Data Availability

The authors confirm that the data supporting the findings of this study are available within the article and its supplementary materials, and may be shared upon request.
