# Supplementary material for: Role of some structural features in EPS from microalgae stimulating collagen production by human dermal fibroblasts
Source: Bioengineered. 2023 Sep 12;14(1):2254027. doi: 10.1080/21655979.2023.2254027 (PMC10498797; doi:10.1080/21655979.2023.2254027)
Supplement: Supplemental Material [file KBIE_A_2254027_SM8070.docx]

**Supplementary material**

| **A)** | 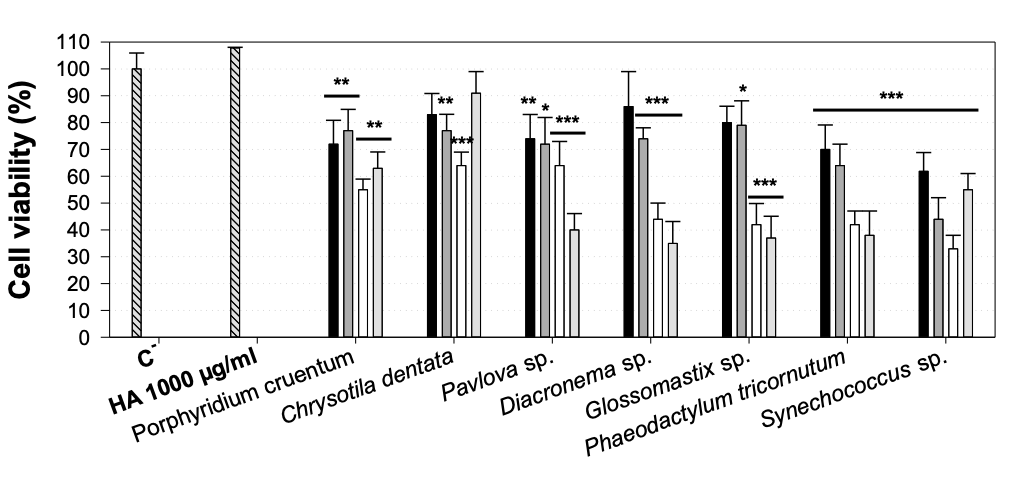 |
| --- | --- |
| **B)** | **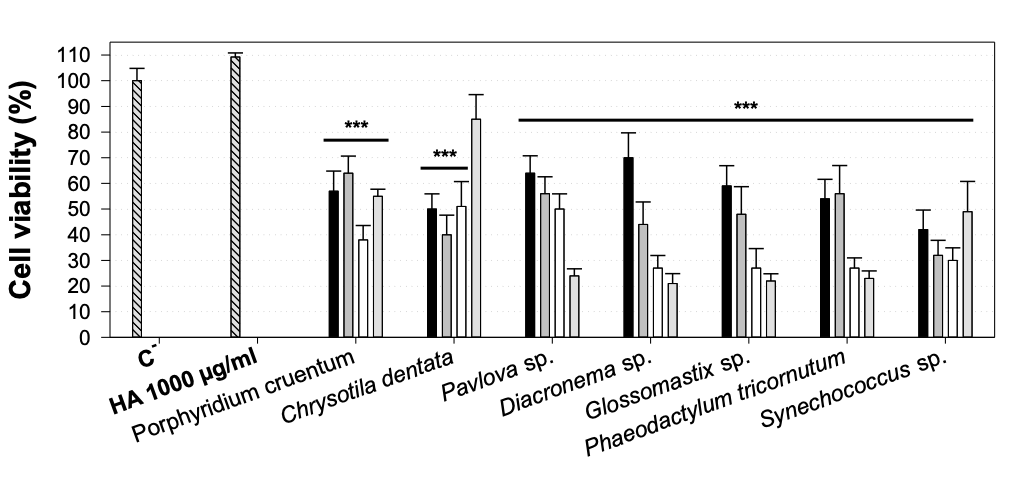** |

**Fig. S1.** Effect of the 7 microalgae EPS, at 100 µg/ml under their native () and depolymerized forms obtained after high pressure pre-treatment (HP-PT: ) and solid acid-catalyzed hydrolysis in batch (B-dep: ) or recycle fixed-bed (RFB-dep: ) systems, on fibroblasts CDD-1059Sk (**A**) and CDD-1090Sk (**B**) viability *in vitro*. Hyaluronic acid at 1000 µg/ml (HA: ) was used as a positive control. Results are expressed as the percentage of cell viability compared to the negative control (C^-^: ). Significant differences between values obtained with samples and negative control are indicated by * (p <0.05), ** (p <0.01) and *** (p <0.001): n=24, N=3.

| **A)** | 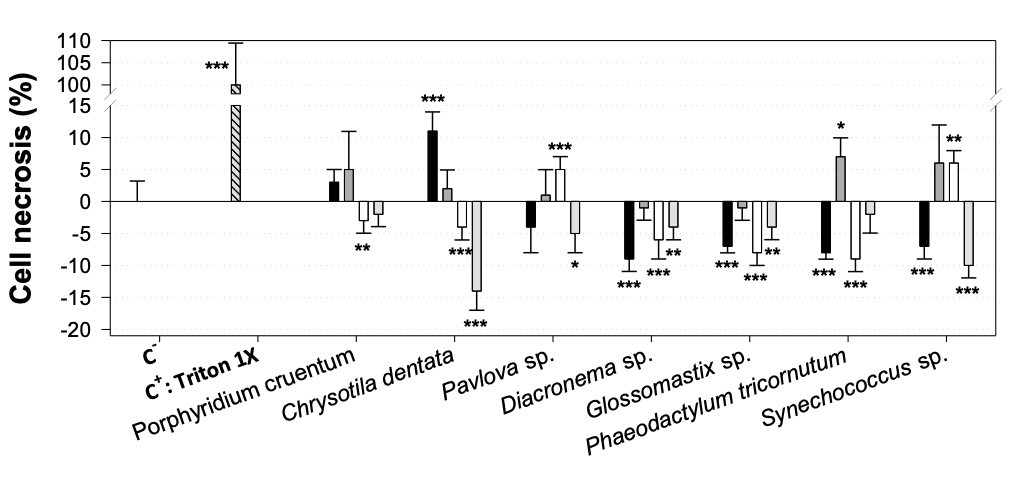 |
| --- | --- |
| **B)** | 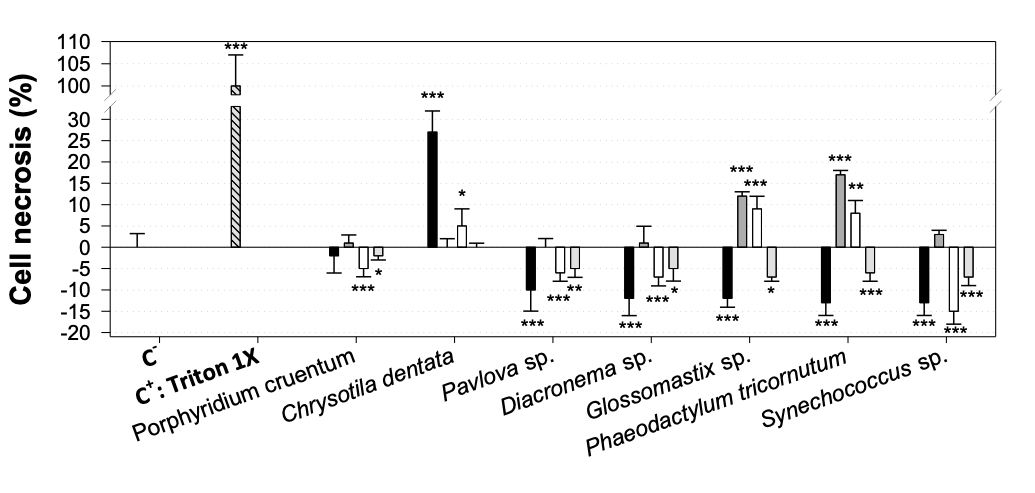 |

**Fig. S2.** Effect of the 7 microalgae EPS, at 100 µg/ml under their native () and depolymerized forms obtained after high pressure pre-treatment (HP-PT: ) and solid acid-catalyzed hydrolysis in batch (B-dep:) or recycle fixed-bed (RFB-dep: ) systems, on fibroblasts CDD-1059Sk (**A**) and CDD-1090Sk (**B**) necrosis *in vitro*. Triton 1X was used as a positive control (). Results are expressed as the percentage of cell necrosis compared to the negative control (C^-^). Significant differences between values obtained with samples and negative control are indicated by * (p <0.05), ** (p <0.01) and *** (p <0.001): n=8.

| **A)** | 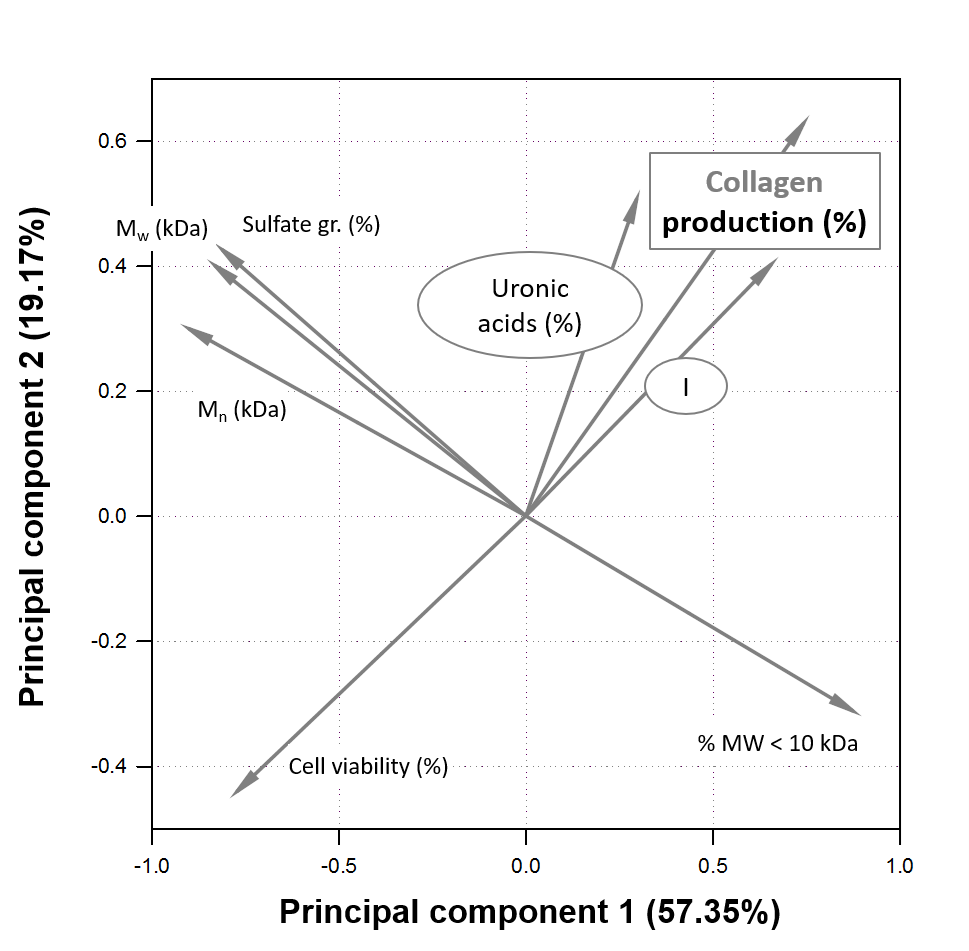 | **B)** | 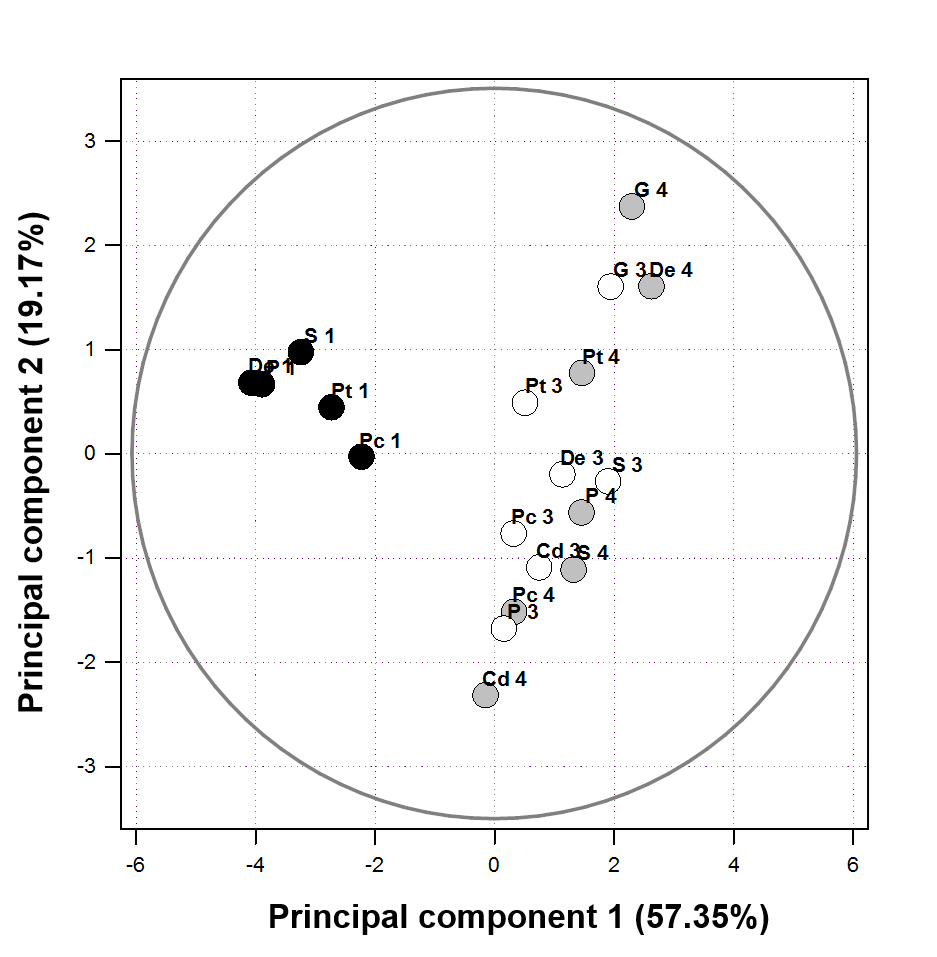 |
| --- | --- | --- | --- |

**Fig. S3.** Principal component analysis (PCA) of the effect of the 7 microalgae EPS under their native and depolymerized forms obtained after high pressure pre-treatment (HP-PT) and solid acid-catalyzed hydrolysis in batch (B-dep) or recycle fixed-bed (RFB-dep) reactor systems, on the collagen production by fibroblasts CDD-1090Sk (**A**: loadings; **B**: scores): EPS from *P. cruentum* (Pc), *C. dentata* (Cd), *Pavlova* sp. (P), *Diacronema ennorea* (De), *Glossomastix* sp. (G), *P. tricornutum*; (Pt) and *Synechococcus* sp. (S), under their native (1), B-dep (3) and RFB-Dep (4) forms.
